# Bitter Apple Pulp‐Derived Porous Carbon with Rich Oxygen Functionalities for High‐Performance Zinc‐Ion Storage

**DOI:** 10.1002/smll.202502071

**Published:** 2025-05-19

**Authors:** Himanshu Gupta, Hem Kanwar Rathore, Manoj Kumar, Prashanth W. Menezes, Debasish Sarkar

**Affiliations:** ^1^ Department of Physics Malaviya National Institute of Technology Jaipur Jaipur Rajasthan 302017 India; ^2^ Defence Laboratory DRDO Jodhpur Rajasthan 342011 India; ^3^ Department of Material Chemistry for Catalysis Helmholtz‐Zentrum Berlin für Materialien und Energie Albert‐Einstein‐Str. 15 12489 Berlin Germany; ^4^ Department of Chemistry, Metalorganics and Inorganic Materials Technical University of Berlin Straße des 17 Juni 135. Sekr. C2 10623 Berlin Germany

**Keywords:** bitter apple pulp, efficient zinc‐ion storage, high surface area activated carbon, KOH‐activation strategy, long durability, plant‐based biomass

## Abstract

Enhancement of the energy‐power performance of aqueous zinc‐ion hybrid supercapacitors (ZIHSCs) relies on the development of high‐performance carbon‐based cathode materials. Porous carbon derived from plant‐based biomasses is particularly attractive due to its rich surface functionalities, high specific surface area (SSA), tunable porosity, cost‐effectiveness, and environmental sustainability. Here, bitter apple pulp (BAP) is explored as a green precursor to realize a novel activated carbon for ZIHSC applications. KOH activation at 900 °C results in a carbon (BAPC1‐900) with an exceptionally high SSA of 3254 m^2^ g^−1^, pore volume of 1.8 cm^3^ g^−1^, and rich oxygen functionalities (C─O, C─OH) significantly outperforming the non‐activated counterpart (64 m^2^ g^−1^, 0.016 cm^3^ g^−1^). Raman spectroscopy reveals that the high SSA and oxygen content facilitate the simultaneous adsorption/desorption of Zn^2+^ and desorption/adsorption of SO_4_^2‐^ ions, ensuring an impressive Zn‐ion energy density of 162 Wh kg^−1^ within a potential window of 0–1.8 V in an aqueous ZnSO_4_ electrolyte for the optimized BAPC1‐900‐based cathode. Besides, the electrode demonstrates excellent reversibility over 50 000 cycles, retaining over 95% capacity retention. This study highlights the efficacy of pulp‐based biomass‐derived porous carbon and KOH‐activation chemistry in maximizing the zinc‐ion storage potential of carbon‐based cathodes for next‐generation energy storage.

## Graphical abstract

An exceptionally high‐surface‐area porous carbon derived from the pulp of bitter apple (*Citrullus colocynthis*) is used as cathode material in zinc‐ion hybrid supercapacitors (ZIHSCs) to achieve an excellent zinc‐ion storage performance over a long duration.

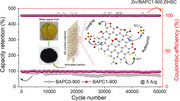

## 1 Introduction

The depletion of crude oil reserves and growing public awareness of its detrimental effects on the environment, such as toxic gas emissions and global warming, are driving increased interest in renewable energy sources.^[^ ^1^ ^]^ However, energy storage is necessary to ensure an effective, continuous, reliable, and reasonably priced energy supply, considering the intermittent nature of renewable energy sources. Among various energy storage systems (ESSs), two of the most advanced electrochemical technologies are batteries and supercapacitors (SCs).^[^ ^2^ ^]^ Although batteries have achieved significant advancements owing to their high energy density, they still face limitations in power density and cycle life. In contrast, SCs offer high power density (>10 kW kg^−1^) and long cycle life (>100 000 cycles) and thus overthrow batteries in high‐power applications. Their low energy density (within 5–10 Wh kg^−1^) is still a setback.^[^ ^3^ ^]^ Therefore, development of advanced energy storage systems that balance both energy and power density is highly desirable, and therefore, hybrid supercapacitors (HSCs) hold great promise.

An HSC integrates a capacitor‐type electrode (enabling high power density and long‐cycling stability) with a battery‐type electrode (providing high energy density) in aqueous or non‐aqueous electrolytes. Aqueous Zn‐ion hybrid supercapacitor (ZIHSC) is an emerging category of HSCs that has garnered much attention lately.^[^ ^4^ ^]^ These systems offer many advantages, such as good energy‐power combination, promising operational potential range, and an exceptionally high safety level owing to the use of aqueous electrolytes. Specifically, ZIHSCs utilize a reversible Zn^2+^/Zn redox reaction on the Zn metal anode and a capacitive mechanism on the carbon cathode to store energy. The Zn metal anode in ZIHSC has an attractive theoretical capacity of 820 mAh g^−1^ and a redox potential of −0.76 V (vs standard hydrogen electrode), which results in high operation voltage and energy density of ZIHSC.^[^ ^5^ ^]^ Generally, the carbon cathodes in ZIHSCs are characterized by high chemical stability and an energy storage mechanism following fast ion adsorption/desorption, which helps in realizing good rate performance and long cycle life.

In this regard, developing carbon cathode materials with hierarchical porous architecture is always advantageous to enhance storage capacity, as cathode operation always depends on the adsorption/desorption of ions at the electrode/electrolyte interface to generate electric double‐layer capacitance (EDLC).^[^ ^6^ ^]^ The utilisation of biomass‐derived carbon has been widely recommended as a potential cathode for ZIHSCs due to its unique characteristics, such as pore tunability, high specific surface area (SSA), environmental friendliness, cost‐effectiveness, abundance of heteroatoms, and chemical stability.^[^ ^7^ ^]^ As different biomasses have different cell‐tissue structures and characteristics, a variety of biomasses including coconut shells,^[^ ^8^ ^]^ bone glue,^[^ ^9^ ^]^ pencil shavings,^[^ ^10^ ^]^ cotton pulp paper,^[^ ^11^ ^]^ lignin,^[^ ^12^ ^]^ agricultural waste (Areca Catechu sheath),^[^ ^13^ ^]^ Zanthoxylum seed cake,^[^ ^14^ ^]^ poplar wood,^[^ ^15^ ^]^ agricultural biomass waste (solanum melongena),^[^ ^16^ ^]^ glutinous rice,^[^ ^17^ ^]^ rice husk,^[^ ^18^ ^]^ etc., have been explored to produce activated carbons with varying SSA, functionalities and performances as cathode materials. Therefore, the selection and optimization of biomass‐based precursors are of great importance to fully utilize the innate special structure of biomasses and realize active carbon materials with high energy storage capability. From a commercial perspective also, to have a viable HSC with state‐of‐the‐art performance, the source of carbon materials should be abundant and inexpensive, thus substantiating the importance of biomasses as carbon precursors. Keeping this in mind, a suitable choice of biomasses and exploring efficient techniques to create hierarchical porous carbon structures are highly coveted.

Pulp‐based carbon precursors have been demonstrated to produce large SSAs following chemical activation, with SSA of 1775 and 2980 m^2^ g^−1^ reported for carbons derived from cellulose pulp and paper pulp sludge, respectively.^[^ ^19^ ^]^ Herein, for the first time, we have activated bitter apple pulp (BAP) derived hydrochar with KOH at different temperatures to realize a novel carbon structure with an exceptionally high SSA of 3254 m^2^ g^−1^ and a total pore volume of 1.8 cm^3^ g^−1^. Bitter apples (*Citrullus colocynthis*) are widely cultivated worldwide for their medicinal, nutraceutical, and food applications.^[^ ^20^ ^]^ The economic viability of the BAP‐derived carbon is demonstrated in Table S1 (Supporting Information), which suggests its advantages over other already explored biomasses. However, their potential as a carbon precursor in the energy sector remains unexplored. Notably, hydroxyl groups (‐OH) on the activated carbon surface significantly enhance hydrophilicity and improve zinc storage capacity by introducing additional pseudocapacitance through fast Faradaic reactions. Here, the KOH‐activation ensures rich oxygen functionalities in the as‐prepared carbon structures, which is crucial for providing abundant electrochemically active sites, a large electrode/electrolyte contact area, and fast electronic/ion transport kinetics across electrode/electrolyte interface in aqueous ZIHSCs. The assembled device, featuring a Zn anode and BAP‐derived carbon cathode, operates within a voltage window of 0–1.8 V in an aqueous ZnSO_4_ electrolyte and yields excellent energy‐power values while demonstrating extremely fast charge characteristics and outstanding cycle stability over thousands of cycles.

## 2 Results and Discussion

### 2.1 Synthesis, Physical and Chemical Characterization of As‐Prepared Materials

**Figure 1:**
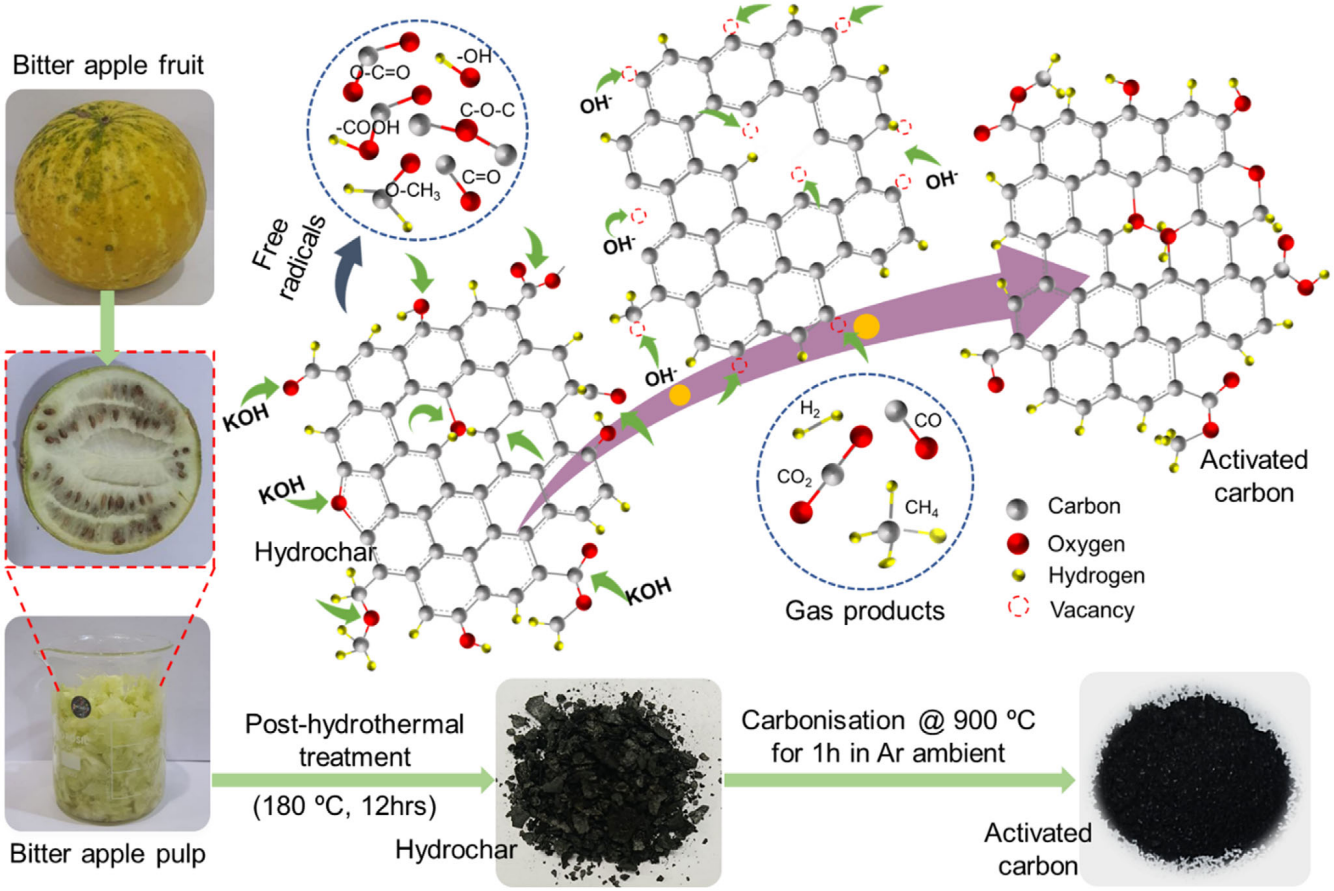
Schematic of the synthesis process of BAP‐derived activated carbon materials.

**Figure** **1** demonstrates the steps for synthesizing BAP‐derived carbon (BAPC) via pre‐carbonization followed by KOH‐activation methods. First, BAP‐derived hydrochar was prepared using hydrothermal treatment of BAP at 180 °C for 12 h in the presence of deionized water (DI). Hydrothermal treatment of BAP includes dehydration, decarboxylation, hydrolysis, polymerization, and condensation reactions to produce a carbonaceous product (hydrochar) with hydrophilic oxygen‐rich structures.^[^ ^21^ ^]^ KOH‐activated carbons achieve high porosity and large SSA through synergistic and comprehensive actions of chemical, physical activation, and carbon lattice expansion by the metallic K intercalation. However, reaction pathways and activation mechanisms vary based on the activation factors (e.g., KOH concentration, activation temperature, etc.), as well as the reactivity of carbon sources. The activation parameters and carbon sources significantly impact the pore microstructure and surface chemistry of activated carbon, and hence, impact its performance in energy storage. First, to investigate the effect of temperature on the KOH activation process, the prepared hydrochar and activating agent (KOH) were mixed together at a fixed KOH/hydrochar weight ratio of 1:1, and then heated at different temperatures. Besides temperature variation, the KOH/hydrochar ratio was also varied to optimize the material's performance. The resulting activated carbon samples were washed, neutralized, and dried for further analyses. Now, we propose the possible chemical reactions during the KOH activation process as follows. First, KOH reacts with the active O‐containing species (─OH, C─O, C═O, O─C═O, and ─COOH) in hydrochar, removing many of these functional groups as free radicals (─OH, C─O, C═O, O─C═O, ─COOH and ─O─CH_3_) and producing vacancies (as shown in Figure 1). At the same time, some parts of KOH react with C─C and C─H groups, etching carbon fragments and releasing abundant protons (H^+^) to generate pores and vacancies. These vacancies are then filled by OH⁻ from KOH, leading to the formation of new O‐containing functional groups, and stabilizing the vacancies. In the meantime, KOH undergoes transformation into K_2_CO_3_ via the reactions between KOH and O‐containing species/carbon fragments at 400–700 °C and is further transformed into K_2_O at higher temperatures, accompanied by the release of metallic K and various gaseous products (detailed discussion is given in Supporting Information). Pores in the as‐prepared activated samples are generated by the etching of carbon framework through chemical reactions between different potassium intermediates and carbon, referred to as chemical activation. Pores, especially micropores, are further generated during the degassing of different volatile species like H_2_O, CO_2_, etc., produced during the chemical activation, and is termed as the physical activation process. Carbon lattices can also expand as a result of the intercalation of metallic K effectively into the interlayers of carbon lattices during activation. The expanded carbon lattices cannot revert to their original form once the intercalated metallic K and other K‐based compounds are removed through washing. As a result, a high microporosity required for significant improvement of SSA and pore volume is generated. So, these processes convert hydrochar into highly porous carbon material.^[^ ^22^ ^]^

**Figure 2:**
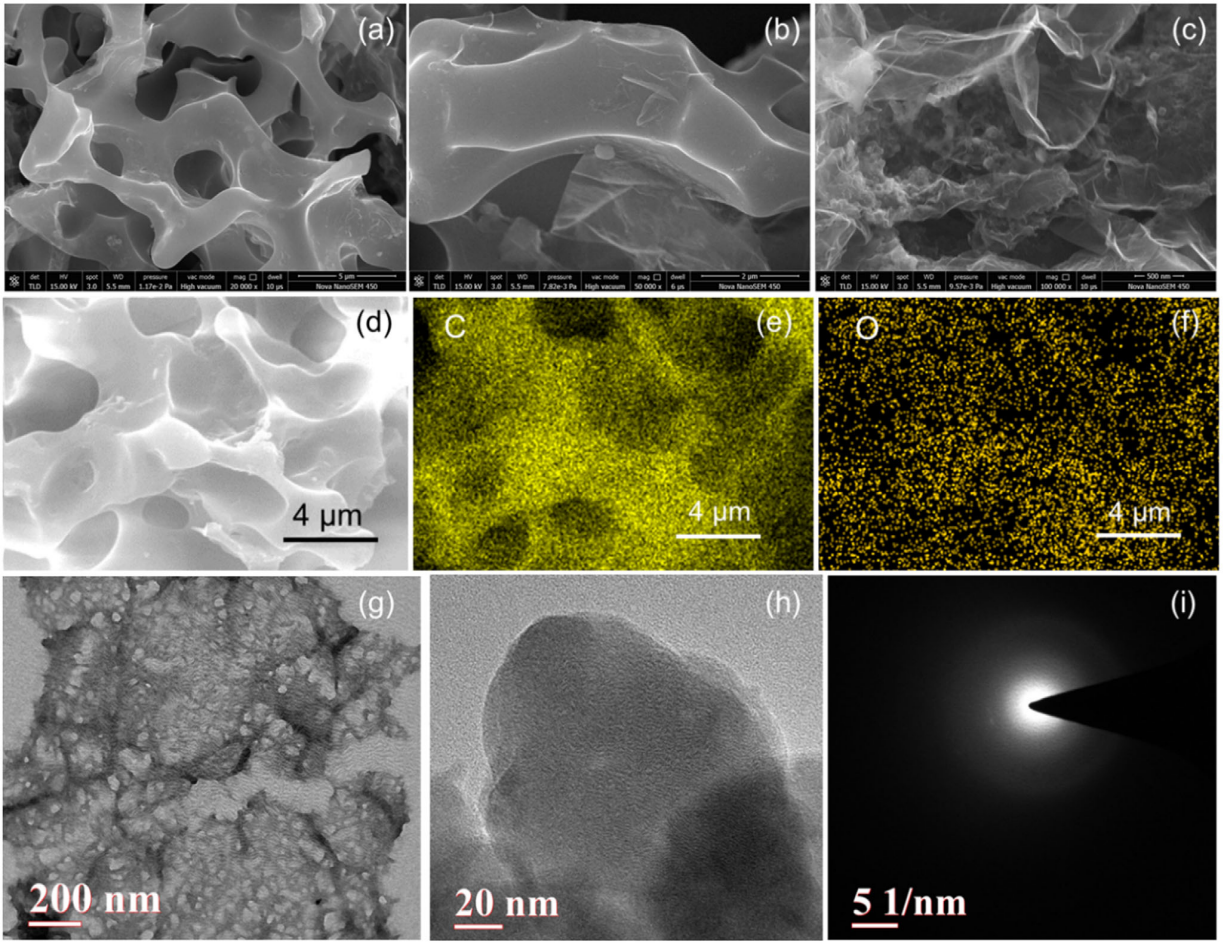
Morphological features of KOH‐activated optimized BAPC1‐900 sample: a–c) FESEM images. d) EDS mapping region and mapping of e) C, f) O. g,h) high‐resolution TEM images. and i) the SAED pattern.

The microstructure of pristine (BAPC0‐900) and KOH‐activated samples was investigated by field‐emission scanning electron microscope (FESEM). The FESEM images of all samples at different resolutions are demonstrated in Figure S1 (Supporting Information) and **Figure** **2**a–c. The microstructure of inactivated BAPC0‐900 is of a solid, irregular sheet‐like structure with no discernible pores (Figure S1, Supporting Information), while the activated BAPC1‐700, BAPC1‐800, and BAPC1‐900 show porous structure credited to the etching process during KOH activation step. However, the microstructure of BAPC3‐900 is completely collapsed and destroyed because of the deep etching in the presence of high KOH content. BAPC1‐900 demonstrated a highly porous structure, as can be seen in Figure 2a–c. Pores in BAPC1‐900 were generated due to the etching process facilitated by KOH with degassing of different volatile moieties at higher temperatures, as represented by Equations S1–S5 (Supporting Information). In Figure 2d–f, an investigation of elemental composition and elemental distribution using Energy‐dispersive X‐ray spectroscopy (EDS) reveals that the BAPC1‐900 sample has only carbon (92.2%) and oxygen (7.65%) as constituents, resulting from abundant carbohydrates in BAP (Table S2, Supporting Information). Moreover, a homogeneous distribution of C and O elements throughout the porous structure could be observed in Figure 2e,f. Transmission electron microscopy (TEM) images of inactivated BAPC0‐900 shown in Figure S2 (Supporting Information) also confirm a thick sheet‐like structure with no visible pores even at higher magnifications. However, the TEM image in Figure 2g suggests that the BAPC1‐900 was actually composed of ultrathin carbon sheets with predominantly disordered structures, the contrast being typical of highly micro and mesoporous structures. The presence of such abundant pores meant that the sample had a large pore volume and high SSA. The high‐resolution TEM (HRTEM) image in Figure 2h again shows the amorphous nature of the material due to a very low degree of ordering and is also supported by the selected area electron diffraction (SAED) pattern in Figure 2i.

**Figure 3:**
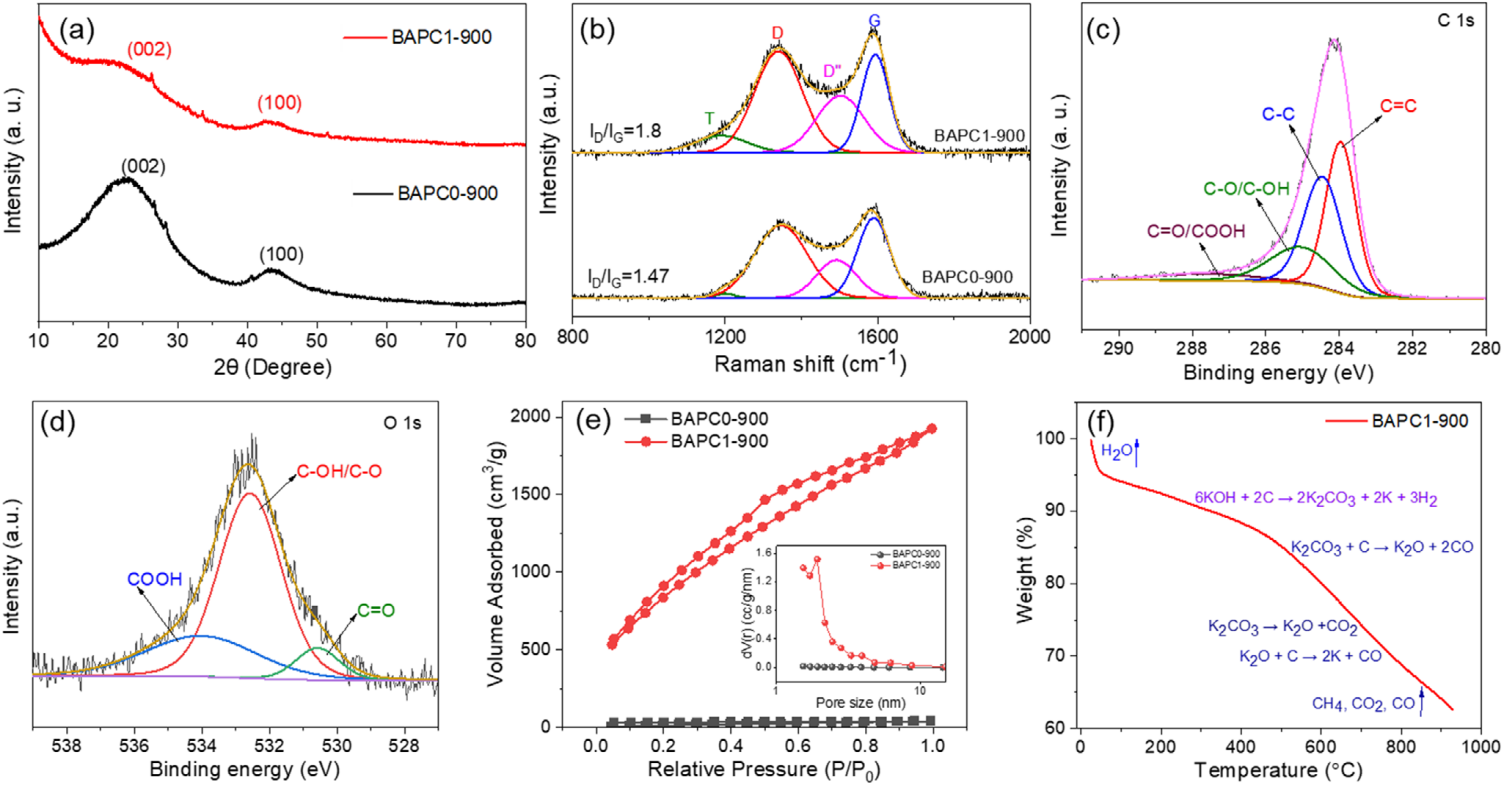
a) XRD spectra of BAPC0‐900 and BAPC1‐900 samples. b) comparison of Raman spectra of BAPC0‐900 and BAPC1‐900 samples. high‐resolution XPS spectra of c) C 1s, d) O 1s for BAPC1‐900. e) N_2_ adsorption‐desorption isotherms (inset shows the pore size distribution graphs) of both the samples, and f) TGA of BAPC1‐900 sample.

The XRD and Raman spectroscopy were used to analyze the BAP‐derived carbon microstructure further. The XRD patterns, as shown in **Figure** **3**a, suggest an overall amorphous nature of BAPC0‐900 & BAPC1‐900 samples characterized by broad and subdued peaks. However, the broad humps at ≈21 and 43° are assigned to the (002) and (100) diffraction planes, respectively, characteristics of some defective graphene domains in the overall amorphous carbon structure. The least intense diffraction peaks of the BAPC1‐900 sample suggest its highly defective nature, which is due to the KOH activation. Moreover, a few small spikes at 26.2, 33, and 51.6° in the XRD spectra are due to some potassium bioproduct residue still remaining after the washing process. Raman spectra for both pristine and activated samples in Figure 3b exhibit four split peaks at ≈ 1200, 1340, 1500, and 1590 cm^−1^, corresponding to T, disordered (D), D″, and graphitic (G) bands, respectively. The T and D″ bands can be ascribed to the defects/heteroatoms.^[^ ^23^ ^]^ The ratio of integrated intensities of D and G bands (I_D_/I_G_) are 1.47 and 1.8 for the pristine (BAPC0‐900) and BAPC1‐900 carbons, respectively, which indicate a higher degree of defectiveness in the activated sample and is credited to the KOH activation strategy creating ample pores and facilitating the formation of defects and disorder in carbon structure through chemical etching reactions. Such defects are good because they help in increasing ion storage capacity by providing additional ion‐binding sites even at higher charge/discharge currents.

The presence of different oxygen‐containing functional groups in the carbon samples was validated using the XPS technique. Figure S3a,d (Supporting Information) depict XPS survey spectra of the BAPC0‐900 and BAPC1‐900 samples, suggesting the presence of only C and O elements. In Figure S3b (Supporting Information), the high‐resolution XPS spectrum of C1s for BAPC0‐900 is deconvoluted into four different peaks corresponding to graphitic carbon C═C (284.4 eV, 55.6%), defective carbon structure C─C (285.2 eV, 21.8%), C─O/C─OH (285.6 eV, 17.6%) and C═O/COOH (289.7 eV, 4.8%) species. However, in Figure 3c, deconvolution of the high‐resolution C1s spectrum for BAPC1‐900 also resulted four peaks corresponding to graphitic carbon C═C (284 eV, 38.5%), defective carbon structure C─C (284.4 eV, 35.3%), C─O/C─OH (285 eV, 20%) and C═O/COOH (287.5 eV, 6.1%) species.^[^ ^24^ ^]^ It is noteworthy that defective carbon species have increased by almost 13% after the KOH activation process, which is in good agreement with the Raman results. Similarly, the deconvoluted O1s XPS spectrum of the pristine sample (Figure S3c, Supporting Information) revealed C═O (531.6 eV, 47%), C─OH/C─O (533.1 eV, 28%), and COOH (534.95 eV, 25%) bonds. Interestingly, for the KOH activated BAPC1‐900 sample, deconvolution of the O1s spectrum (Figure 3d) discloses C═O (530.57 eV, 8%), C─O/C─OH (532.57 eV, 66%), and COOH (534.02 eV, 26%) bonds, which also suggests a higher relative content of hydroxyl species in the activated carbon structure.^[^ ^25^ ^]^ A comparative Fourier transform infrared spectroscopy (FT‐IR) study shown in Figure S4 (Supporting Information) confirmed the enhancement of hydroxyl group content after activation, where a relatively high, intense broad peak (≈3400 cm^−1^) for the BAPC1‐900 sample was evident. Besides, after KOH activation, weakening of the C─H and C═C stretching vibration bands centered around 1392 and 1633 cm^−1^, respectively, suggested carbon etching to produce ample pores in the material, which is also in agreement with the reduction in C─C/C═C bond content in BAPC1‐900 per the XPS analyses. However, the band at 1116 cm^−1^ can be assigned to C─O stretching vibrations in alcohols, phenols, ether, or ester groups, while the band located at 612 cm^−1^ is due to the O─H out‐of‐plane bending vibrations in both samples.^[^ ^26^ ^]^ To validate the efficacy of the KOH activation, N_2_‐adsorption‐desorption isotherms were used to assess the pore structure, and corresponding isotherms for BAPC0‐900 and BAPC1‐900 samples are shown in Figure 3e. As per IUPAC, the isotherms can be classified as type‐IV with an H3 type hysteresis loop. A steep N_2_ uptake at P/P_0_ < 0.2, a hysteresis loop in the range of 0.45–0.65, and a further enhancement in N_2_ uptake at P/P_0_ > 0.8 could be observed, indicating the coexistence of micropores and mesopores in BAPC1‐900 sample. The Brunauer‐Emmett‐Teller (BET) SSA of pristine BAPC0‐900 was only 64 m^2^ g^−1^ (Table S3, Supporting Information), indicating its limited application potential. However, KOH activation significantly enhanced the SSA to an exceptionally high value of 3254 m^2^ g^−1^ for the BAPC1‐900 sample, demonstrating the effectiveness of the pulp‐based precursors and the activation strategy used. Interestingly, as revealed by the t‐plot method, the contribution of micropore SSA is more than 50% (1910 m^2^ g^−1^) of the total BET SSA of BAPC1‐900. Moreover, the Barrett‐Joyner‐Halenda (BJH) pore size distribution analysis, as shown in the inset of Figure 3e, reveals that BAPC1‐900 has a total pore volume of 1.82 cm^3^ g^−1^ (with a micropore contribution of 0.93 cm^3^ g^−1^), significantly higher than 0.016 cm^3^ g^−1^ observed for the BAPC0‐900 sample. The details pore characteristics of these samples are summarized in Table S3 (Supporting Information). These results confirm that KOH activation of BAP produces both meso‐ and micropores in abundance within the synthesized carbon structure, which would be efficient in facilitating electrolyte ion penetration deep inside the electrode material to enhance ion storage capacity at fast charging rates. The thermal behavior of the BAPC1‐900 was studied by thermogravimetric analysis (TGA), and the mass loss as a function of temperature is shown in Figure 3f. The initial mass loss of BAPC1‐900, up to 5%, occurred at low temperatures (25 < *T* < 80 °C) due to moisture loss and the desorption of physically adsorbed water on the material. The decomposition of KOH and the pyrolysis process arose at a temperature range of 100–500 °C with a net weight loss of ≈15%. The next significant weight loss phase began at 500 °C. Weight loss at such a high temperature can be attributed to the decomposition of different functional groups found in BAPC1‐900, including phenols, esters, ethers, quinones, etc.^[^ ^27^ ^]^ The FTIR analysis (Figure S4, Supporting Information) further supported the presence of these functional groups. Additionally, the carbonization/activation process at higher temperatures releases gaseous volatiles such as CH_4_, CO, CO_2_, and aldehydes, contributing to the observed weight loss.^[^ ^22^, ^28^ ^]^

### 2.2 Electrochemical Properties of BAPC Electrodes

**Figure 4:**
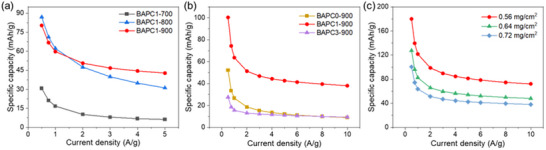
Specific capacity versus current density graphs for different carbon samples: a) activated at different temperatures with KOH/biochar ratio fixed at 1. b) activated at 900 °C with different KOH/biochar ratios, and c) mass loading variation of BAPC1‐900 as cathode active material.

Detailed electrochemical properties of the as‐prepared carbon samples were studied in aqueous ZIHSC half‐cells. The ZIHSC contains a Zn foil anode, as‐prepared carbon materials loaded onto stainless steel (SS) as the cathode, and a 2 m ZnSO_4_ aqueous electrolyte. In a process to optimize the performance of carbon materials, **Figure** **4**a–c compares their cathodic performance within a stable operating potential window of 0–1.8 V (vs Zn^2+^/Zn) following different measurement protocols. First, we compared materials activated at different activation temperatures (BAPC1‐700, BAPC1‐800, and BAPC1‐900) to determine the optimal activation temperature. As shown in Figure 4a, BAPC1‐900 exhibited the best performance in terms of specific capacity and rate capability among all three samples, suggesting 900 °C as the optimal activation temperature for BA pulp‐derived carbons. Next, the KOH/biochar ratio was varied to prepare BAPC0‐900 (pristine), BAPC1‐900 (1:1), and BAPC3‐900 (3:1) samples, and their electrochemical performance was evaluated (Figure 4b). Again, BAPC1‐900 delivered the best performance among all three samples. At high temperatures (>700 °C), reaction by‐products (K_2_CO_3_, CO_2,_ and K_2_O, etc.) were reduced in CO and metallic K by carbon contributing to the formation of abundant pores and a high SSA. Thus, an optimum KOH content results in an optimized porous structure of the carbon material and a superior charge storage performance. Further electrochemical measurements were carried out on BAPC1‐900 to optimize its mass loading density in the cathode, as shown in Figure 4c. The results indicate that the specific capacity decreased with increased active material loading, and for 0.56 mg cm^−2^ loading density, we achieved the highest specific capacity (180 mAh g^−1^) with good rate performance. Lowering loading density further was not pursued due to its non‐feasibility from a practical application perspective.

**Figure 5:**
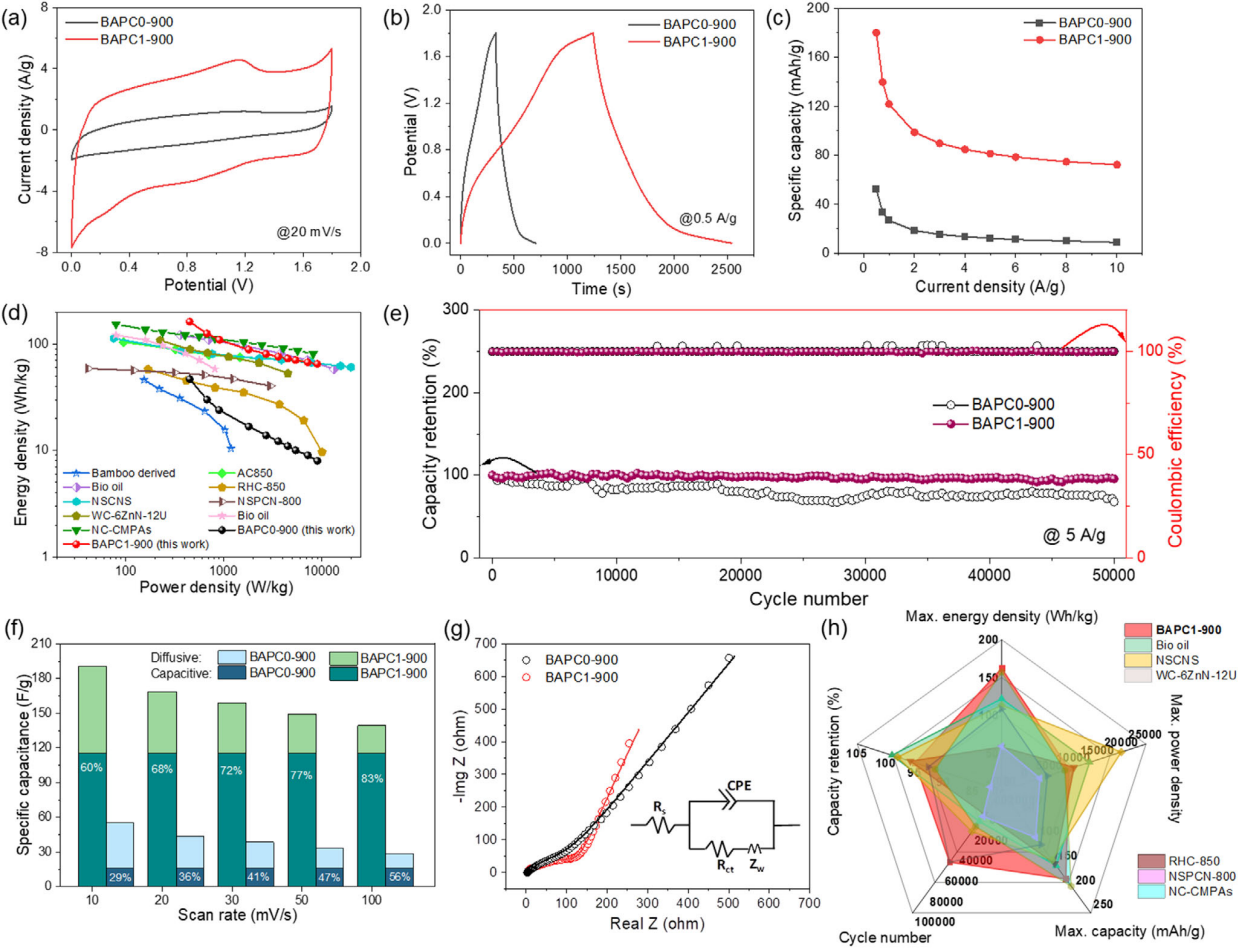
Electrochemical characteristics of as‐prepared pristine BAPC0‐900 and activated BAPC1‐900 as ZIHSC cathode: comparison of a) CV curves. b) GCD profiles. c) Specific capacities at different current rates. d) its energy‐power densities compared with other recent literature reports. e) Cycling stability. f) Capacitive and diffusive contributions to the total capacity for both samples. g) Comparative EIS plots (inset shows equivalent circuit utilized for fitting). and h) Radar plot comparing the electrochemical performance of BAPC1‐900 and other recently published carbon‐based cathodes in ZIHSCs.

**Figure** **5**a compares the CV curves of BAPC1‐900 (with a loading density of ≈0.56 mg cm^−2^) and BAPC0‐900 (pristine, not activated) at a scan rate of 20 mV s^−1^. CV curves exhibited a quasi‐rectangular shape, which typically characterizes ZIHSC behavior. Additionally, the CVs displayed prominent humps in both anodic and cathodic scans, reflecting Faradaic redox reactions between oxygen‐containing functional groups and electrolytic ions, as well as Zn plating/striping (Zn ↔ Zn^2+^+2e^−^) reactions. Furthermore, the Zn//BAPC1‐900 ZIHSC exhibits a significantly higher current density than Zn//BAPC0‐900 ZIHSC, indicating a higher capacity for storing ions on the cathode surface. The Zn//BAPC1‐900 cell maintained a consistent quasi‐rectangular shape of CVs even when scan rates were increased from 10 to 200 mV s^−1^, indicating good rate capability and fast charge transfer kinetics (Figure S5a,b, Supporting Information). Quantitatively, the specific capacities at a scan rate of 10 mV s^−1^ were calculated to be 27 and 92 mAh g^−1^ for BAPC0‐900 and BAPC1‐900 samples, respectively (Figure S5c, Supporting Information). This substantial improvement in BAPC1‐900 can be attributed to the significantly larger SSA and pore volume.

In Figure 5b, a comparative GCD analysis of the cathode materials demonstrates similar triangular‐shaped GCD curves, suggesting their comparable charge storage mechanism. However, the sloping discharge profile, especially at lower voltages (<0.2 V vs Zn^2+^/Zn), can be attributed to the charge storage on the heteroatoms (O) and at defect sites, a phenomenon commonly observed in similar systems.^[^ ^29^ ^]^ The superior performance of BAPC1‐900 is evident from its prolonged discharge profile, which resulted in a specific capacity of 180 mAh g^−1^ at a current density of 0.5 A g^−1^, significantly higher than 52 mAh g^−1^ obtained for the BAPC0‐900 electrode (Figure 5c). Figure S6 (Supporting Information) shows GCD curves of the cathode materials at different current densities to better understand their charge storage kinetics and performance. As the GCD analyses reveal, the BAPC1‐900 could deliver specific capacities as high as 180, 140, 122, 99, 90, 85, 81, 78, 75, and 72 mAh g^−1^ at 0.5, 0.75, 1, 2, 3, 4, 5, 6, 8, and 10 A g^−1^, with almost 40% capacity retention at the highest current density (Figure S6a, Supporting Information). In contrast, BAPC0‐900 could only show a 16% rate capability at 10 A g^−1^. All these superior performances of BAPC1‐900‐cathode‐based ZIHSC could only be attributed to the synergy between its high SSA (3254 m^2^ g^−1^) with almost 1920 m^2^ g^−1^ of micropore area and high oxygen‐containing functional groups, which provide ample reaction sites for physical and chemical adsorption/desorption of electrolytic ions, thus enhancing the capacity of the electrode materials. Besides, the large pore volume (of ≈1.8 cm^3^ g^−1^) of BAPC1‐900 would also facilitate ion diffusion throughout the electrode and hence, produce good capacity even during fast charge/discharge rates. Moreover, the vacancies and defects in the carbon structure could also attract electrolytic ions and contribute to the total capacity of the electrode material. Therefore, a higher degree of defectiveness of the BAPC1‐900, as validated by the XPS and Raman investigations should help in enhancing its storage capacity by providing additional ion‐binding sites.

Ragone plot in Figure 5d presents the energy versus power density graphs for BAPC0‐900 and BAPC1‐900 cathodes, in comparison with other developed carbon‐based cathodes for ZIHSCs. It can be observed that the Zn//BAPC1‐900 ZIHSC reached a maximum energy density of 162 Wh kg^−1^ at a power density of 450 W kg^−1^ while delivering 65 Wh kg^−1^ of energy density at a maximum power density of 9 kW kg^−1^ (calculated based on the active material mass loading in the cathode). Such remarkable energy‐power delivery capability of the Zn//BAPC1‐900 significantly outperforms Zn//BAPC0‐900 and surpasses most recently reported ZIHSCs with carbon‐based cathodes, including bamboo‐derived carbon,^[^ ^30^ ^]^ AC850,^[^ ^31^ ^]^ Bio‐oil,^[^ ^32^ ^]^ RHC‐850,^[^ ^33^ ^]^ NSCNS,^[^ ^34^ ^]^ NSPCN‐800,^[^ ^35^ ^]^ WC‐6ZnN‐12U,^[^ ^15^ ^]^ and NC‐CMPAs.^[^ ^36^ ^]^ Besides, Zn//BAPC1‐900 ZIHSC exhibits outstanding cycling stability and ultra‐long cycle life, retaining 95.7% of its initial capacity after 50 000 GCD cycles at a current density of 5 A g^−1^, highlighting its robust electrode architecture. Moreover, the coulombic efficiency was also retained at 100% (Figure 5e), demonstrating excellent reversibility of electrode reactions. In contrast, BAPC0‐900//Zn ZIHSC could retain only 68% of its initial capacity after 50 000 cycles with a ≈100% coulombic efficiency, indicating that chemical activation is crucial for the long‐term stability of electrode materials. Finally, Table S4 (Supporting Information) provides a performance comparison between BAPC1‐900‐based ZIHSC and the most recently reported carbon‐based ZIHSCs, validating BAPC1‐900 as a state‐of‐the‐art cathode material.

Utilizing CVs at various scan rates allows for an in‐depth examination of the charge storage kinetics of the BAPC1‐900 cathode material. This investigation assesses the relative contribution of capacitive and diffusion‐controlled processes to the overall stored charge in the electrode material. Generally, capacitive contribution comes from “EDLC” and rapid Faradaic reactions at the electrode‐electrolyte interface, referred to as “pseudocapacitance”. In contrast, the diffusion of ions from the electrolyte within the bulk of the electrode material contributes to the diffusion‐controlled storage process. The detailed analysis method can be found in the Supporting Information. The logarithms of peak currents (*i*) and scan rates (*v*) are linearly related, and their relationship can be expressed in terms of *b*‐values, where *b* = 1 represents capacitive‐type surface‐dominated reactions, while *b* = 0.5 refers to diffusion‐controlled redox reactions.^[^ ^5^, ^37^ ^]^ As illustrated in Figure S7a,b (Supporting Information), the BAPC1‐900 electrode exhibits *b*‐values nearly equal to 1, which suggests the dominance of the capacitive processes. To further quantify capacitive and diffusion‐controlled charge storage contributions, the Dunn method was employed for BAPC0‐900 and BAPC1‐900 cathode, with results plotted at different scan rates in Figure 5f.^[^ ^38^ ^]^ With decreasing scan rates, capacitive contribution decreases, or diffusion contribution increases, as ions get sufficient time to diffuse deep into the cathode material at slow scan rates. Notably, for BAPC1‐900, the capacitive contribution increases from 60% at 10 to 83% at 100 mV s^−1^, implying that ion adsorption/desorption and Faradaic redox processes primarily regulate the charge storage mechanism. On the other hand, the capacitive contribution in BAPC0‐900 increases from 29% at 10 mV s^−1^ to 56% at 100 mV s^−1^, significantly lower than that of BAPC1‐900. In Figure S8a,b (Supporting Information), the capacitive‐controlled current is plotted against voltage at a scan rate of 100 mV s^−1^, which shows a significantly high capacitive contribution (83%) for BAPC1‐900 compared to BAPC0‐900. These results confirm that the superior charge storage capability of BAPC1‐900 is facilitated by rapid electrochemical kinetics occurring predominantly on its surface. Therefore, an optimized activation process can effectively reconstruct the pore structure and incorporate abundant redox‐active functional groups, both critical for achieving state‐of‐the‐art Zn ion storage performance with carbon‐based electrodes.

Electrodes’ kinetics were further evaluated using electrochemical impedance spectroscopy (EIS). Figure 5g represents the EIS spectra for both BAPC0‐900 and BAPC1‐900 samples. The EIS spectra comprised a semicircle in the high‐frequency region that provides the charge transfer resistance and a sloping line in the low‐frequency region characterizing the diffusion of Zn ions. To better understand the electrochemical processes, the EIS data were fitted with an equivalent circuit model (inset of Figure 5g) to estimate the series resistance (*R*_s_) and charge transfer resistance (*R*_ct_). BAPC1‐900 displayed lower *R*_s_ and *R*_ct_ values as compared to BAPC0‐900, indicating fast reaction kinetics and rapid charge transfer of the former (Table S5, Supporting Information). The diffusion of electrolyte ions was reflected by the linear region of the Nyquist plot in the low‐frequency region and can be assessed properly according to the equation: *ω* = 2*πf* and *Z* = *R* + *σω* ^−1/2^, where *ω* is the angular frequency, *f* is the corresponding test frequency, and *Z* is a real part of the impedance. The Warburg coefficient (*σ*) is a measure of the ion‐diffusion kinetics, with a smaller *σ* value indicating faster ion diffusion.^[^ ^39^ ^]^ The *σ* value of BAPC1‐900 was calculated to be 35.36, which is much smaller than 122.6 obtained for the BAPC0‐900 (Figure S8c, Supporting Information), suggesting that BAPC1‐900 facilitates ion transport owing to its large hierarchical porous structure and hydrophilic nature due to the presence of oxygen functionalities, thus contributing to superior Zn‐ion storage performance.^[^ ^40^ ^]^ The Radar plot in Figure 5h summarizes some of the critical performance matrices of BAPC1‐900‐based ZIHSC and compares those with other recently reported carbon‐based ZIHSCs, where BAPC1‐900‐based ZIHSC excels in almost all performance areas.

### 2.3 Energy Storage Mechanism of Aqueous ZIHSC

**Figure 6:**
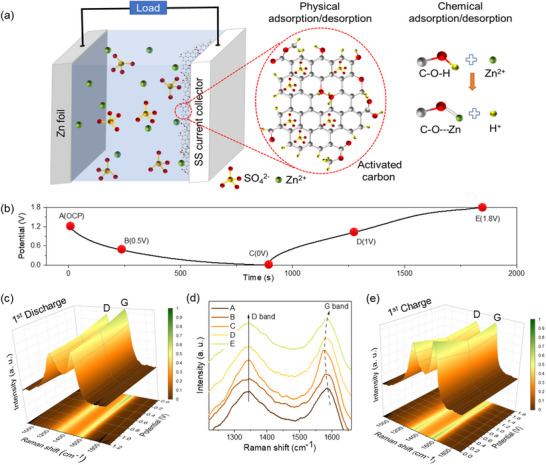
a) Schematic mechanism of charge storage on BAPC1‐900 carbon cathode. b) First GCD profile at 0.5 A g^−1^ with different SOCs marked with red spheres. c) Raman spectrum during 1st discharging step, d) Raman shift of G band positions, and e) Raman spectrum during 1st charging step.

A schematic of the ZIHSC and its related charge storage mechanism on a carbon‐based cathode is illustrated in **Figure** **6**a. The primary charge storage mechanism of the activated carbon‐based cathode involves physical adsorption/desorption of SO_4_^2−^ ions on the carbon surface; the higher the surface area, the greater the capacity. Additionally, Zn^2+^ ions chemically bond to the C─O groups, replacing H in C─OH bonds, which contributes to extra redox pseudocapacitance and, consequently, an increase in specific capacity.^[^ ^41^ ^]^ To further investigate its energy storage mechanism, Raman spectroscopy was used to measure the changes in D and G bands of BAPC1‐900‐based cathodes at various states of charge (SOCs). As shown in Figure 6b, five representative points (A, B, C, D, and E) were selected on the first GCD profile of the BAPC1‐900‐based cathode to analyze its structural changes. As illustrated in Figure 6c, when the fresh BAPC1‐900 electrode was discharged from the open circuit potential (OCP, 1.22 V) to the lowest charge state, the intensity of the D and G bands in the Raman spectrum was found to increase gradually. This can be explained in terms of Zn^2+^ adsorption on the BAPC1‐900 electrode, which suppresses phonon energy dissipation due to the stretching of the C─C bonds. The interaction between adsorbed Zn^2+^ and the BAPC1‐900 electrode is also responsible for the red shift in the G‐band of BAPC1‐900 observed in the fully discharged state (C), as shown in Figure 6d.^[^ ^42^ ^]^ Meanwhile, the I_D_/I_G_ ratio (Figure S9a, Supporting Information) decreases with increasing zincation depth, indicating a higher degree of graphitization or lowering of the disorder in carbon structure due to the presence of adsorbed Zn^2+^ ions. However, in the 1st charging stage, desorption of Zn^2+^ was indicated by a gradual reduction in the intensity of the Raman spectra when the BAPC1‐900 electrode was charged from 0 V (C) to 1 V (D) as illustrated in Figure 6e. Subsequently, the intensity of the Raman spectrum increased again as the electrode was further charged to 1.8 V (E), which can be attributed to the adsorption of SO_4_^2−^ ions on the electrode surface as charging progresses.^[^ ^43^ ^]^ Therefore, the charging process is associated with the desorption of Zn^2+^ ions as well as the adsorption of SO_4_^2−^ ions.^[^ ^44^ ^]^ Accordingly, although I_D_/I_G_ ratio increases again during charging (through SOCs C→D→E) (Figure S9a, Supporting Information), the change was not as significant as that observed while discharging (through SOCs A→B→C). However, during 2nd discharging step from 1.8 V (E) to 0 V (G) (Figure S9b,c, Supporting Information), suppression of the G‐band red shift could be observed, which suggests the discharge process was governed by both adsorption and desorption of Zn^2+^ and SO_4_^2−^ ions, respectively. Owing to the capacitance contributions from SO_4_^2−^ and good electronic conductivity of the as‐synthesized activated carbon, the Zn//BAPC1‐900 supercapacitor has demonstrated high storage capacity with excellent reversibility over tens of thousands of charge/discharge cycles.

## 3 Conclusion

We have presented BAP as a novel green precursor and developed a facile method to produce value‐added carbon materials with exceptionally high SSA and pore volume. By optimizing KOH/hydrochar weight ratio of 1:1 and activating at 900 °C (BAPC1‐900), we achieved a SSA as high as 3254 m^2^ g^−1^ along with a large pore volume of 1.8 cm^3^ g^−1^. Moreover, KOH treatment introduced abundant redox‐active oxygen functionalities into the final carbon structure. It is hypothesized that such traits would be highly beneficial toward zinc‐ion storage in ZIHSCs. Notably, the optimized BAPC1‐900 carbon‐based cathode attained a state‐of‐the‐art specific zinc‐ion storage capacity of 180 mAh g^−1^ and an energy density of 162 Wh kg^−1^ in aqueous ZnSO_4_ electrolyte, outperforming most of the recently reported activated carbon‐based ZIHSC cathodes. Raman analyses indicate that although the 1st cycle discharge capacity is associated with the adsorption of Zn^2+^ ions mainly, the coexistence of Zn^2+^ desorption/adsorption and SO_4_^2‐^ adsorption/desorption processes makes the successive charge‐discharge cycles reversible. Consequently, the ZIHSC with the optimized cathode demonstrated impressive cycle stability with 95% retention after 50 000 cycles, along with 100% coulombic efficiency. This study provides valuable insights into the zinc‐ion storage mechanism in BAP‐derived activated carbon and establishes it as a promising electrode material for high‐performance, affordable, and safe ZIHSCs. Furthermore, we envision that beyond its potential in the renewable energy storage sector, BAP‐derived high surface area carbon could also find applications in many other emerging fields, including water treatment and gas storage, offering significant commercial potential in the future.

## 4 Experimental Section

### Materials Synthesis

Bitter apple fruits were purchased from the market of Jaipur, Rajasthan. Pulp was separated from the fruit to prepare porous carbons through a two‐step approach, including hydrothermal pre‐carbonization and activation, as shown in Figure 1. First, BAP‐derived hydrochar was prepared using hydrothermal treatment at 180 °C for 12 h in the presence of DI. For activation, the prepared hydrochar and activating agent KOH were mixed together at a KOH/hydrochar weight ratio of 1:1. This mixture was placed in an alumina boat and heated in a tube furnace to 700, 800, and 900 °C at a heating rate of 5 °C min^−1^ under Ar ambient and held at the final temperature for 1 h, after which the sample was allowed to cool down under Ar flow. Then, the mass ratio of KOH and hydrochar was varied by taking different mass ratios (KOH/char = 1 and 3) and activating them at a temperature of 900 °C for 1 h in Ar atmosphere. The pristine sample was also prepared via direct carbonization of hydrochar at 900 °C. Finally, all these prepared samples were washed with 1 m HCl to remove metal residuals and washed thoroughly with DI water until the pH was close to 7. The obtained black powder is dried at 80 °C overnight to obtain the final porous carbon. Final prepared samples are named by the general term BAPCX‐T, where BAPC is bitter apple pulp‐derived carbon, X is KOH/biochar ratio, and T is the activation temperature; BAPC0‐T represents a pristine sample.

### Preparation of BAP‐Derived Carbon Materials Electrode

Using the slurry‐casting technique, the BAPC materials‐based electrodes were fabricated on a 1 × 1 cm^2^ stainless steel (SS) foil substrate. Acetylene black (Alfa Aesar, ≥99%) (10% in mass), BAPC materials (80% in mass), and polyvinylidene fluoride (PVDF, Sigma‐Aldrich) (10% in mass) were properly mixed with a few drops of N‐methyl‐2‐pyrrolidone (NMP, Alfa Aesar, ≥99%) solvent to prepare the slurry. The working electrodes were fabricated by coating the slurry on SS foils and drying it overnight at 80 °C in the air oven. Weighing the SS foil substrate both before and after the slurry casting process allows us to determine the mass loading of active material.

### Techniques for Structural, Morphological, and Electrochemical Characterization

The prepared materials' crystalline structure and phase purity were examined using the XRD technique (Panalytical X Pert Pro). The materials were morphologically examined using techniques such as TEM (the Tecnai G2 20 S‐TWIN), HRTEM, and FESEM, FEI (Nova Nano FESEM 450). The EDS technique was employed when examining various constituent elements. XPS (ESCA+ Omicron Nano Technology) and FT‐IR (Perkin Elmer) were utilized to analyze their chemical surroundings. The degree of graphitization of the as‐synthesized materials was examined using Raman spectroscopy (Renishaw). BET surface area of the as‐prepared samples was assessed using N_2_ adsorption/desorption techniques (Nova Touch LX2 gas sorption analyzer, Quantachrome Instruments), and pore size distributions were obtained following the BJH model. TGA was performed using a Perkin Elmer STA6000 instrument in an inert environment with a N_2_ flow rate of 20 mL min^−1^ and varying the temperature from 25 to 900 °C at a constant heating rate of 5 °C min^−1^.

The room temperature electrochemical characteristics of ZIHSCs were investigated, which comprised Zn foil anode and BAP‐derived carbon materials‐based cathodes in 2 m ZnSO_4_ electrolyte. The electrodes were dipped in the electrolyte for over three hours before electrochemical investigation. Galvanostatic charge–discharge (GCD) measurements at different current densities and cyclic voltammetry (CV) at various scan rates were used in the investigation. These experiments provided several electrochemical performance characteristics, including stability, energy‐power density, and rate performance. Electrochemical impedance spectroscopy (EIS) was performed at the open circuit potential with an AC field amplitude of 5 mV and a frequency range from 100 kHz to 10 mHz.

## Conflict of Interest

The authors declare no conflict of interest.

## Supporting information



Supporting Information

## Data Availability

The data that support the findings of this study are available from the corresponding author upon reasonable request.
