# Supplementary material for: Bitter Apple Pulp‐Derived Porous Carbon with Rich Oxygen Functionalities for High‐Performance Zinc‐Ion Storage
Source: Small. 2025 May 19;21(30):2502071. doi: 10.1002/smll.202502071 (PMC12306392; doi:10.1002/smll.202502071)
Supplement: Supplementary file 1 — Supporting Information [file SMLL-21-2502071-s001.docx]

Supporting Information for

**Bitter Apple Pulp-Derived Porous Carbon with Rich Oxygen Functionalities for High-Performance Zinc Ion Storage**

*Himanshu Gupta, Hem Kanwar Rathore, Manoj Kumar, Prashanth W. Menezes*, Debasish Sarkar**

H. Gupta, H. K. Rathore, M. Kumar, D. Sarkar

Department of Physics, Malaviya National Institute of Technology Jaipur, Rajasthan 302017, India. E-mail: [debasish.phy@mnit.ac.in](mailto:debasish.phy@mnit.ac.in)

H. K. Rathore

Defence Laboratory, DRDO, Jodhpur, Rajasthan 342011, India

P. W. Menezes

Department of Material Chemistry for Catalysis, Helmholtz-Zentrum Berlin für Materialien und Energie, Albert-Einstein-Str. 15, 12489 Berlin, Germany.

E-mail: [prashanth.menezes@helmholtz-berlin.de](mailto:prashanth.menezes@helmholtz-berlin.de)

P. W. Menezes

Department of Chemistry: Metalorganics and Inorganic Materials, Technical University of Berlin, Straße des 17 Juni 135. Sekr. C2, 10623 Berlin, Germany

E-mail: [prashanth.menezes@mailbox.tu-berlin.de](mailto:prashanth.menezes@mailbox.tu-berlin.de)

**Computation of specific capacity, energy and power densities for ZIHSC-**

The specific capacitance (C_s_, measured in F/g) and specific capacity (C, measured in mAh/g) is determined using the discharge data of the galvanostatic charge-discharge curves (GCD) through the application of the following equation:

$$Specific capacitance: C_{s}=\frac{i*\Delta t}{m*\Delta V}$$

$$Specific capacity:C=\frac{C_{s}*\Delta V}{3.6}$$

Where i (A) is the current, Δt (s) is the discharge time for discharge window ΔV (V), and m (g) is the active mass of material.

The energy density (E, Wh/kg) and power density (W/kg) of ZIHSC are calculated using these equations:

$$Energy density: E=\frac{1}{2*3.6}C_{s}{\Delta V}^{2}$$

$$Power density: P=\frac{3600*E}{\Delta t}$$

Where, C_s_ (F/g) is the specific capacitance, ΔV (V) is the operating potential window, and Δt (s) is the discharge time.

**KOH activation process:**

The following reactions mainly occur during the KOH activation process-

$6KOH +2C\to2K_{2}CO_{3} +2K+3H_{2}$ (S1)

$K_{2}CO_{3} +C \to K_{2}O +2CO$ (S2)

$K_{2}CO_{3}\to K_{2}O +CO_{2}$ (S3)

$CO_{2} +C \to2CO$ (S4)

$K_{2}O +C \to2K +CO$ (S5)

During KOH activation, initially, K_2_CO_3_ forms at about 400 °C, as shown in eqn (S1). The KOH is completely consumed at about 700 °C. At temperatures higher than 700 °C, produced K_2_CO_3_ considerably decomposes into CO_2_, CO and K_2_O (eqns (S2) & (S3)); at ~ 800 °C, it completely disappears. Furthermore, at high temperatures (> 700 °C), the resulting CO_2_ can be reduced by carbon to form CO (eqn (S4)), and K_2_O can also be reduced by carbon to produce metallic K (eqn (S5)). ^[1]^

**Table S1**:Estimated overall cost viability of the bitter-apple-pulp derived carbon and compared the same with other available reports-

| **S. No.** | **Biomass Source** | **Availability** | **Derived carbon SSA (m^2^/g)** | **Electrochemical performance** | **Overall Cost viability** | **Ref.** |
| --- | --- | --- | --- | --- | --- | --- |
| 1 | Bitter apple | Seasonal, agricultural waste | 3254 | High (porous, good conductivity) | Low-moderate | **This work** |
| 2 | Sugar beet pulp | Seasonal, eatable | 624 | Low-moderate | Low-moderate | ^[2]^ |
| 3 | Sweet potato | Seasonal, eatable | 3424 | Moderate-high | Moderate | ^[3]^ |
| 4 | Coconut shell | Widely available | 1260 | Moderate-high | Moderate | ^[4]^ |
| 5 | Orange peel | Seasonal, limited | 2160 | Moderate-high | Moderate | ^[5]^ |
| 6 | Tea Waste | Readily available | 911 | Low-moderate | Moderate | ^[6]^ |
| 7 | Rice husk | Seasonal, agricultural waste | 1012 | Low | Low-moderate | ^[7]^ |
| 8 | Teak wood | Tree based | 898 | Low | Low-moderate | ^[8]^ |
| 9 | Corn stalks | Common agriculture waste | 2495 | Moderate | Moderate-high | ^[9]^ |
| 10 | Popcorn derived | Seasonal, abundant agriculture grain | 3301 | Moderate-high | Moderate-high | ^[10]^ |
| 11 | Olive husk | Fruit based waste | 2900 | High | Moderate-high | ^[11]^ |
| 12 | Jute stick | Plant-based | 1370 | Moderate | Moderate | ^[12]^ |

**
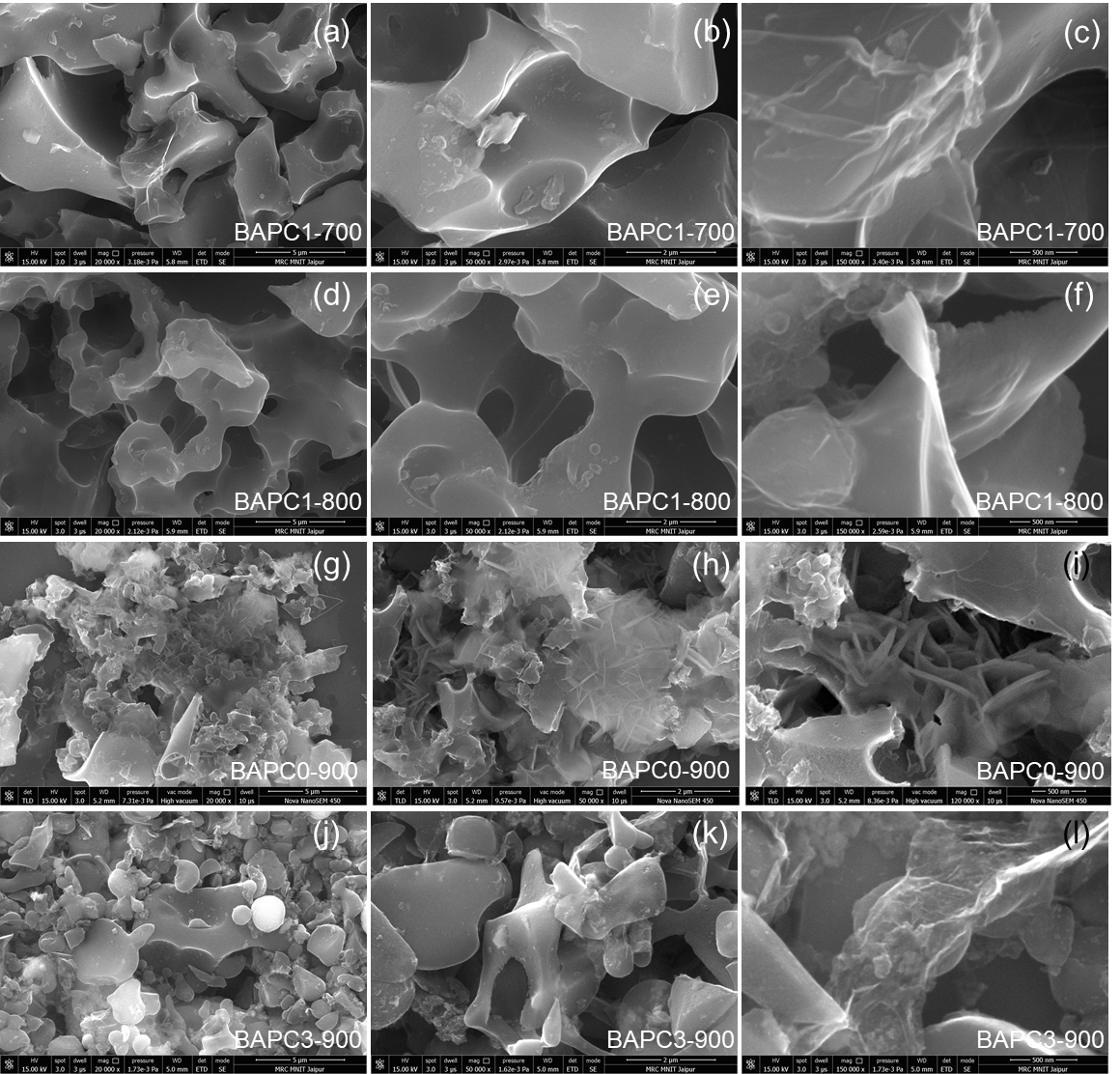
**

**Figure S1.** FESEM images of BAP-derived carbon samples at different resolutions: a-c) BAPC1-700; d-f) BAPC1-800; g-i) BAPC0-900 and j-l) BAPC3-900.

**Table S2:** Atomic concentration of different elements in BAPC1-900 by EDS-

| Elements | Atomic% |
| --- | --- |
| C | **92.22** |
| O | **7.65** |

**
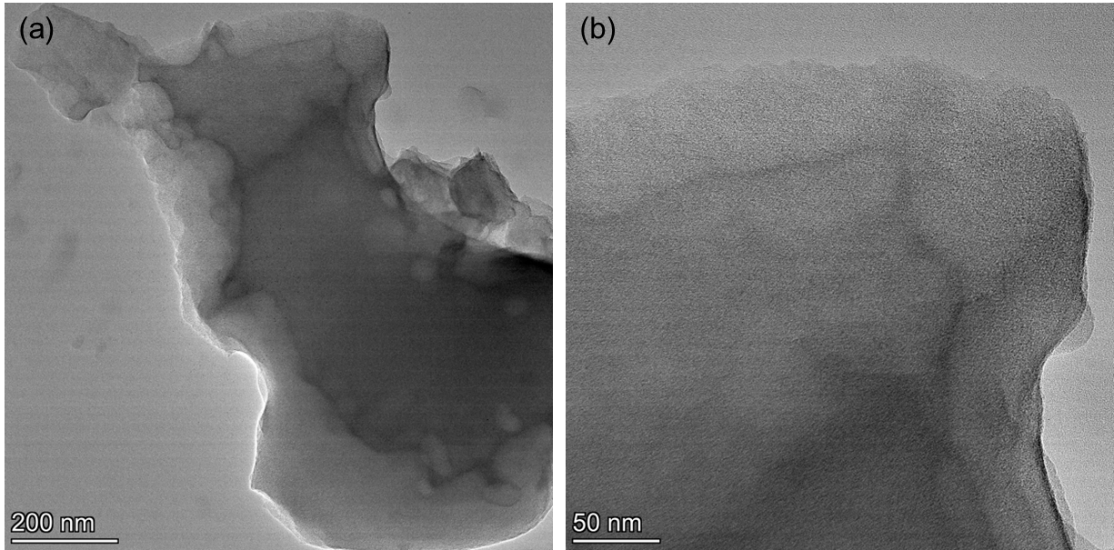
**

**Figure S2.** TEM images of BAPC0-900 samples.

**
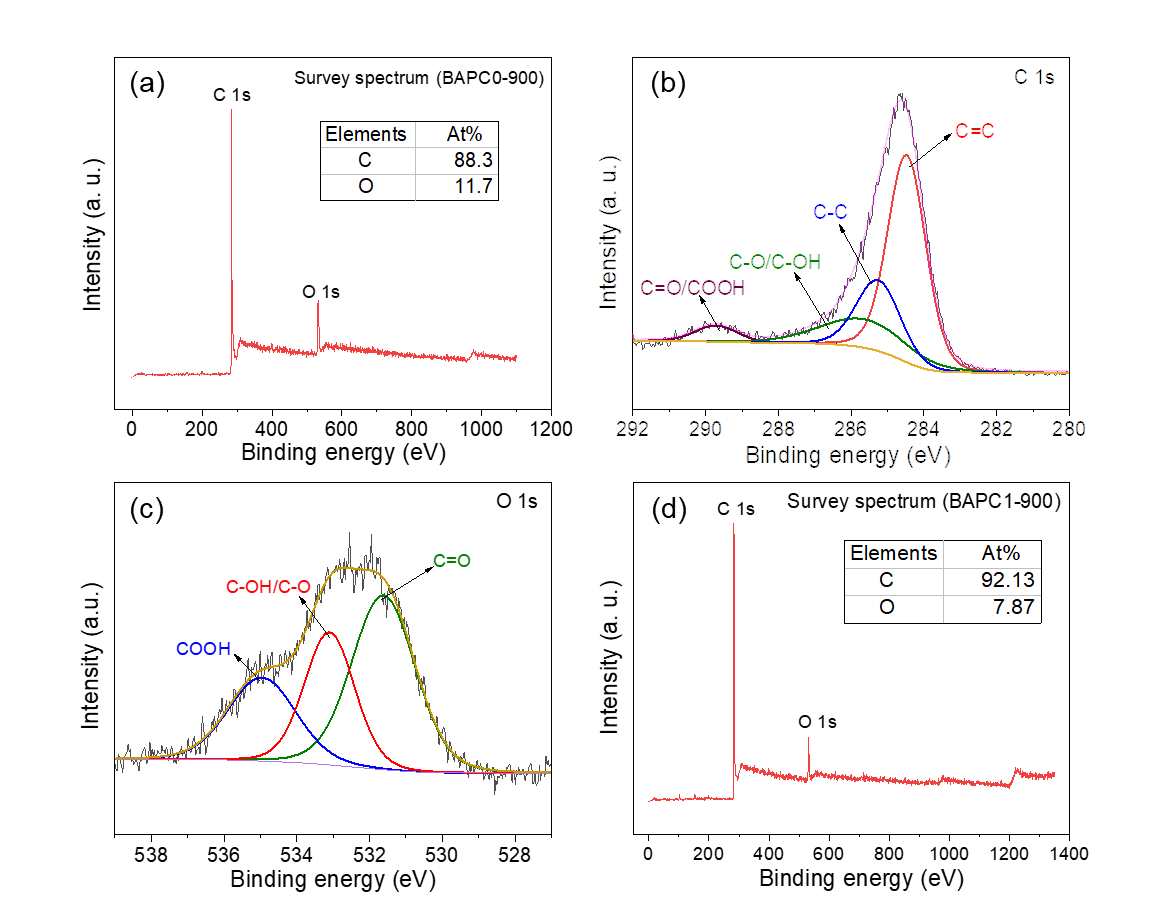
**

**Figure S3.** a) XPS survey spectrum of BAPC0-900, its high-resolution spectrum for b) C 1s, c) O 1s, and d) XPS survey spectrum of BAPC1-900 sample.

**
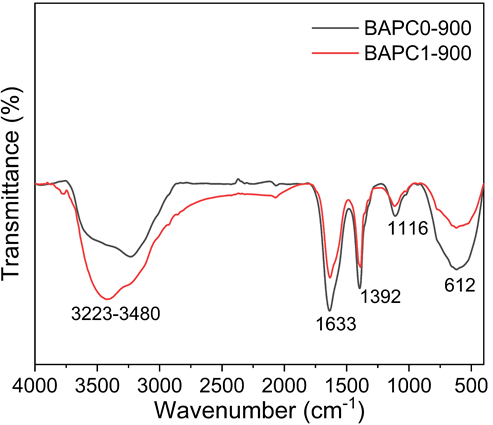
**

**Figure S4.** Comparative FT-IR spectra for the BAPC0-900 and BAPC1-900 samples.

**Table S3:** Pore characteristics of BAPC0-900 and BAPC1-900 samples-

| **Sample** | **BET SSA (m^2^/g)** | **Total pore volume (cm^3^/g)** | **Micropore SSA (m^2^/g)^[a]^** | **Micropore volume (cm^3^/g)^[a]^** | **Meso-/Macropore area (m^2^/g)^[b]^** | **Meso-/Macropore volume (cm^3^/g)^[b]^** |
| --- | --- | --- | --- | --- | --- | --- |
| BAPC0-900 | 64 | 0.016 | 30 | 0.01 | 34 | 0.006 |
| BAPC1-900 | 3254 | 1.82 | 1910 | 0.93 | 1344 | 0.89 |

[a] Calculated using t-plot method.

[b] Calculated by subtracting the total pore volume/BET SSA from the micropore volume/micropore area.

**
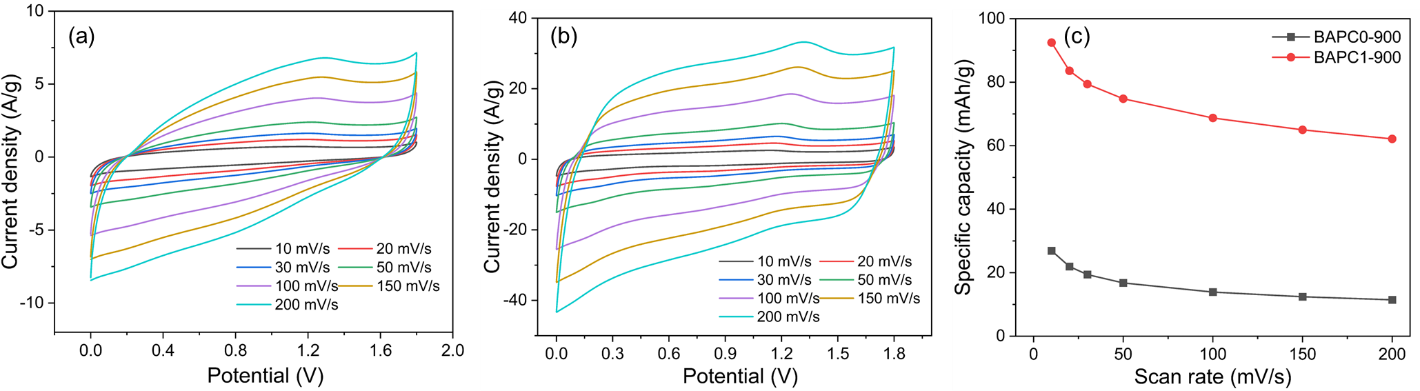
**

**Figure S5.** CV plots at different scan rates for a) BAPC0-900, b) BAPC1-900, and c) comparison of specific capacity at different scan rates.


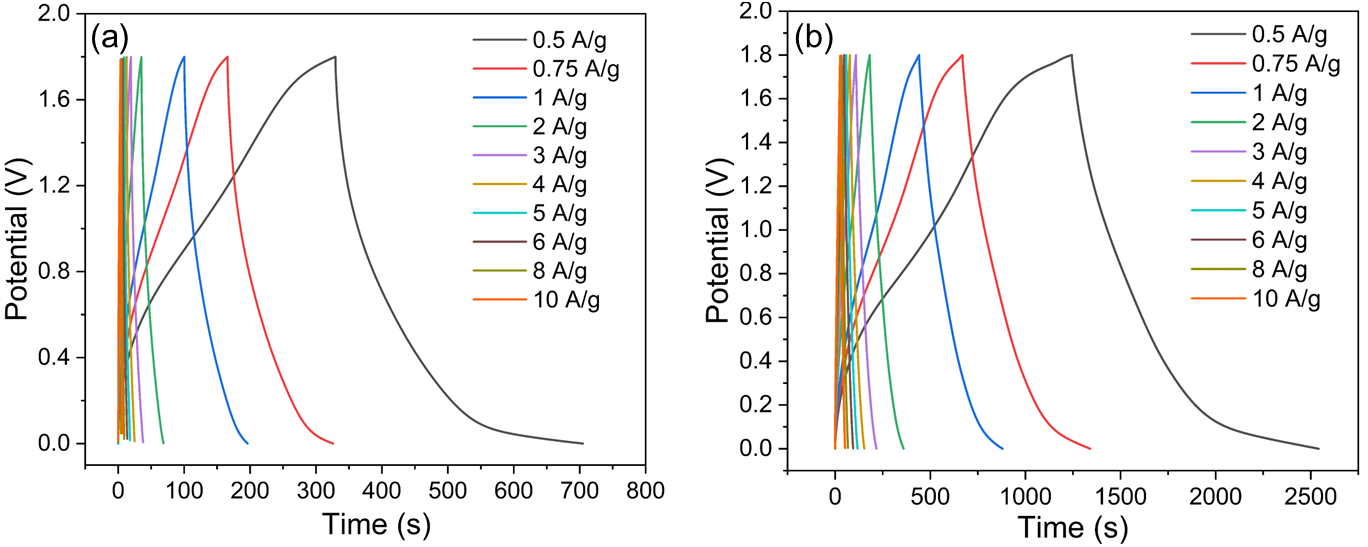
 **Figure S6**. GCD curves at different current densities for a) BAPC0-900 and b) BAPC1-900.

**Deconvoluting capacitive and diffusive charges from CV as a function of scan rate-**

The peak current in the CVs and scan rates obey the following power law (eqn(S6)):

$i=av^{b}$ (S6)

Here, *a* & *b* are two adjustable parameters. A process that stores charge through diffusion-controlled is indicated by *b* = 0.5. while a value of *b* = 1 refers to ‘capacitive’ processes. The slope of the straight-line plot of log(*i*) versus log(*v*) (**Figure S5a**) at different charge-discharge voltages can be used to calculate *b*-values (as shown in **Figure S5b**). Now, due to the difference in scan rate dependence, the contributions of capacitive and diffusive processes can be mathematically separated. Dunn and coworkers detailed the procedure in the literature.^[13]^ To put it succinctly, this technique uses the following equation (eqn (S7)) to simplify the diffusion-controlled and capacitive contributions to the current $i\left( V \right)$ at a given voltage:

$i\left( V \right)=a_{1}v+a_{2}v^{1/2}$ (S7)

The above equation can be rearranged as follows:

$i\left( V \right)/v^{1/2}=a_{1}v^{1/2}+a_{2}$ (S8)

The slopes and intercepts, $a_{1}$and $a_{2}$, can now be determined by plotting the straight lines $i\left( V \right)/v^{1/2}$ vs. $v^{1/2}$ at various voltages. At certain potentials, the current contributions from diffusive ($a_{2}v^{1/2}$) and capacitive ($a_{1}v$) processes can then be determined.^[14]^

**
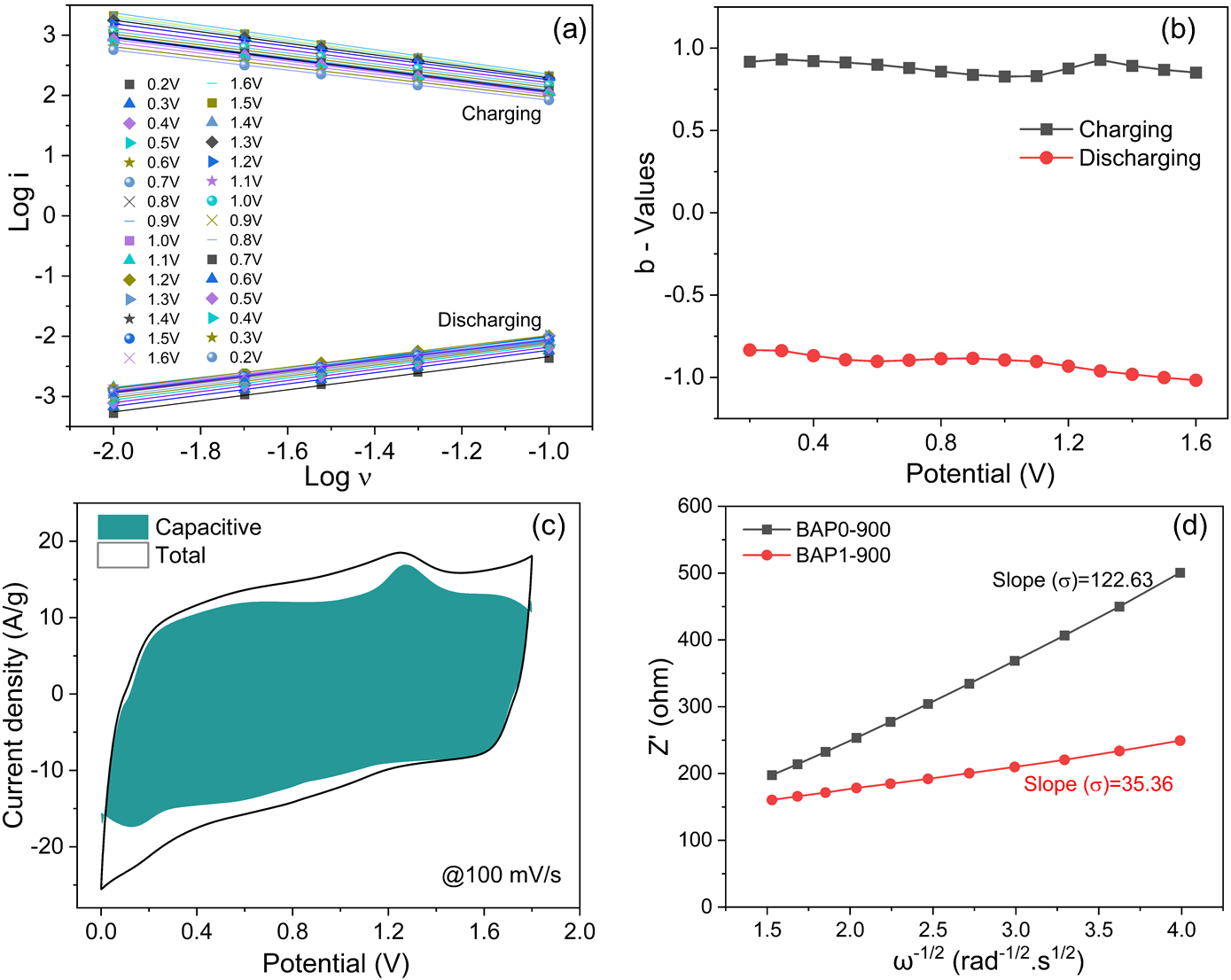
 Figure S7**. For charging and discharging of BAPC1-900 electrode at different voltages: a) log(*i*) versus log(*v*) plot and b) b-values curve.

**
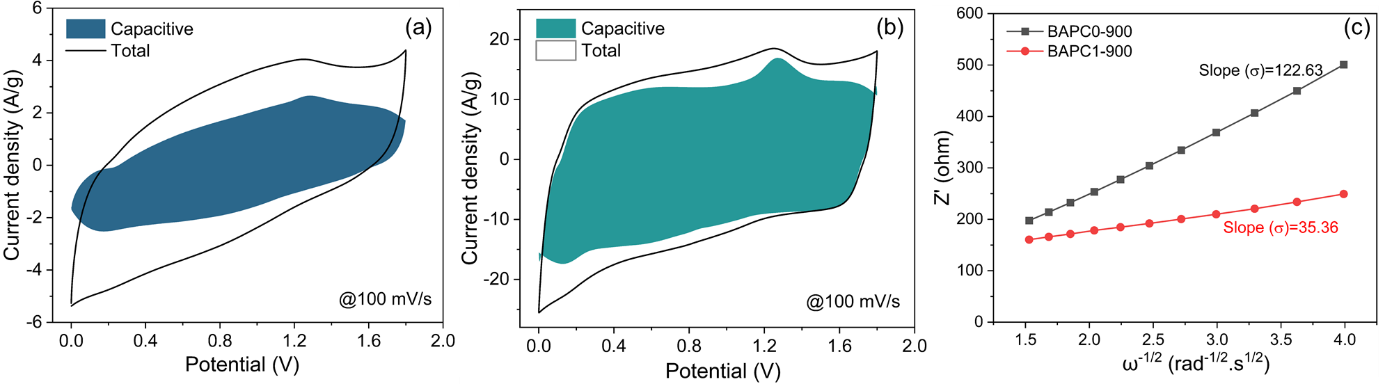
Figure S8**. Voltage variation of capacitive controlled current with respect to the total current measured at 100 mV/s for a) BAPC0-900, b) BAPC1-900 and c) The relationship between the real part of the impedance and low frequencies for both samples.

**Table S4:** Electrochemical performance of different cathode materials in ZIHSCs-

| Cathode material | Electrolyte | Potential window (V) | Capacity (mAh/g) | Energy density (Wh/kg) | Power density (kW/kg) | Ref. |
| --- | --- | --- | --- | --- | --- | --- |
| BAPC1-900 | 2M ZnSO_4_ | 0-1.8 | **180 (0.5A/g)** | **162** | **0.45** | **This work** |
| BAPC0-900 | 2M ZnSO_4_ | 0-1.8 | **52 (0.5A/g)** | **47** | **0.45** | **This work** |
| S, N-CNC | 2M ZnSO_4_ | 0-1.8 | 165.5(1A/g) | 148.9 | 0.9 | ^[15]^ |
| PCNF-4 | 1M ZnSO_4_ | 0.1-1.7 | 177.7(0.5A/g) | 142.2 | 0.4 | ^[16]^ |
| BCF | 2M ZnSO_4_ | 0.1-1.8 | 133.5 (1 A/g) | 119.7 | 0.89 | ^[17]^ |
| Bio-oil | 1M Zn(CF3SO3)2 | 0.2-1.8 | 153.3 (0.5 A/g) | 119 | 0.095 | ^[18]^ |
| BH-4 | 2M ZnSO_4_ | 0.2-1.8 | 132 (1 A/g) | 117.5 | 0.89 | ^[19]^ |
| PBC-A900 | Zn(CF_3_SO_3_)_2_ | 0.2-1.8 | 142.8 (1 A/g) | 114 | 0.8 | ^[20]^ |
| NSCNS | 2M ZnSO_4_ | 0.2-1.8 | 141.5 (0.1 A/g) | 113.4 | 0.075 | ^[21]^ |
| N, O-HPC | 2M ZnSO_4_ | 0.2-1.8 | 138.5 (0.5 A/g) | 110 | 0.138 | ^[22]^ |
| HNPC | 1M ZnSO_4_ | 0-1.8 | 177.8 (4.2 A/g) | 107.3 | - | ^[23]^ |
| N, S-PCD | 2M ZnSO_4_ | 0.2-1.8 | 133.4 (0.2 A/g) | 106.7 | 0.016 | ^[24]^ |
| AC-850 | 1M ZnSO4 | 0-1.8 | 125.7 (1 A/g) | 104.4 | 0.095 | ^[25]^ |
| NPG | 1M ZnSO_4_ | 0-1.8 | 105.1 (0.5 A/g) | 94.6 | 0.44 | ^[26]^ |
| AC | 2M ZnSO_4_ | 0.2-1.8 | 121 (0.1 A/g) | 84 | - | ^[27]^ |
| PCM-800 | C_6_F_6_O_6_S_2_Zn | 0-1.9 | 113.3 (0.1 A/g) | 64.9 | 0.028 | ^[28]^ |
| NSPCN-800 | 2M ZnSO4 | 0.2-1.8 | 95.2 (0.1 A/g) | 59 | 0.041 | ^[29]^ |
| RHC-850 | 3M  Zn(CF_3_SO_3_)_2_ | 0.1-1.8 | 70.7 (0.2 A/g) | 58 | 0.169 | ^[7]^ |
| ZIGC | Zn(CF_3_SO_3_)_2_ | 0-1.8 | 146.5 (0.5 A/g) | 120 | 0.45 | ^[30]^ |
| HMFC | 2M ZnSO_4_ | 0.2-1.8 | 132 (0.1 A/g) | 95.2 | 0.074 | ^[31]^ |
| N-Ov-NCO@CC | 6M KOH+0.2 Zinc acetate | 1.2-2.0 | 107 (1 A/g) | 50 | 0.3 | ^[32]^ |
| S-MCF-N | Zn(CF_3_SO_3_)_2_ | 0.2-1.8 | 92.4 (0.2 A/g) | 72.4 | 0.15 | ^[33]^ |

**Table S5:** Series and charge transfer resistance for both samples-

| Sample | R_s_ | R_ct_ |
| --- | --- | --- |
| BAPC0-900 | **2.87** | **170** |
| BAPC1-900 | **1.93** | **160** |

**
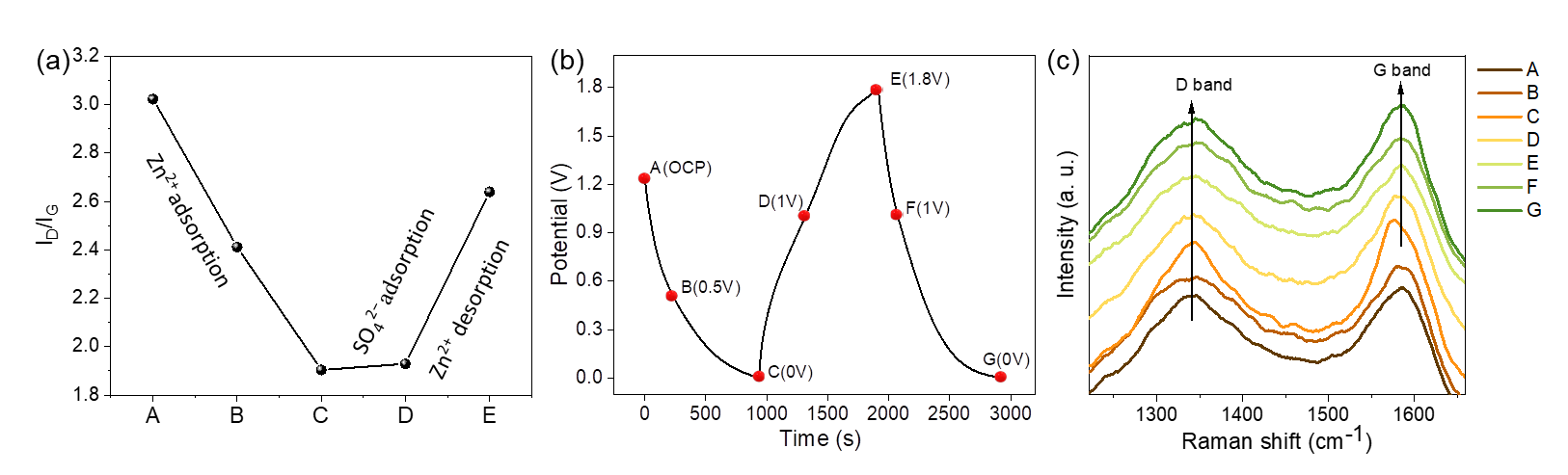
Figure S9.** a) Plot of ratios of integrated intensities (I_D_/I_G_) at different SOCs, b) GCD profile recorded at 0.5 A/g, c) ex-situ Raman spectrum taken at different SOCs during complete charge/discharge processes.

**References**

[1] a) J. Wang, S. Kaskel, *J. Mater. Chem.* **2012**, *22*, 23710; b) W. Chen, M. Gong, K. Li, M. Xia, Z. Chen, H. Xiao, Y. Fang, Y. Chen, H. Yang, H. Chen, *Appl. Energy* **2020**, *278*, 115730.

[2] E. Gür, T. G. Semerci, F. Semerci, *J. Energy Storage* **2022**, *51*, 104363.

[3] H. Hu, G. Wu, *Int. J. Electrochem. Sci.* **2021**, *16*, 210937.

[4] G. Yang, J. Huang, X. Wan, Y. Zhu, B. Liu, J. Wang, P. Hiralal, O. Fontaine, Y. Guo, H. Zhou, *Nano Energy* **2021**, *90*, 106500.

[5] K. Subramani, N. Sudhan, M. Karnan, M. Sathish, *ChemistrySelect* **2017**, *2*, 11384.

[6] X. Song, X. Ma, Y. Li, L. Ding, R. Jiang, *Appl. Surf. Sci.* **2019**, *487*, 189.

[7] Y. Liu, H. Tan, Z. Tan, X. Cheng, *Appl. Surf. Sci.* **2023**, *608*, 155215.

[8] A. Chakraborty, A. Paul, A. Ghosh, N. C. Murmu, T. Kuila, *Energy Storage* **2024**, *6*, e540.

[9] Y. Cao, K. Wang, X. Wang, Z. Gu, Q. Fan, W. Gibbons, J. D. Hoefelmeyer, P. R. Kharel, M. Shrestha, *Electrochim. Acta* **2016**, *212*, 839.

[10] J. Hou, K. Jiang, R. Wei, M. Tahir, X. Wu, M. Shen, X. Wang, C. Cao, *ACS Appl .Mater. Interfaces* **2017**, *9*, 30626.

[11] R. Nasser, J. Tiantian, J.-M. Song, *J. Energy Storage* **2022**, *51*, 104348.

[12] M. M. Mohamed, M. A. Aziz, A. Hussain, Y. P. Hardianto, Z. H. Yamani, *J. Energy Storage* **2024**, *100*, 113635.

[13] a) H.-S. Kim, J. B. Cook, H. Lin, Jesse S. Ko, Sarah H. Tolbert, V. Ozolins, B. Dunn, *Nat. Mater.* **2017**, *16*, 454; b) J. Wang, J. Polleux, J. Lim, B. Dunn, *J Phys. Chem. C* **2007**, *111*, 14925.

[14] a) C. Liu, E. I. Gillette, X. Chen, A. J. Pearse, A. C. Kozen, M. A. Schroeder, K. E. Gregorczyk, S. B. Lee, G. W. Rubloff, *Nat. Nanotechnol.* **2014**, *9*, 1031; b) D. Sarkar, D. Das, S. Das, A. Kumar, S. Patil, K. K. Nanda, D. D. Sarma, A. Shukla, *ACS Energy Lett.* **2019**, *4*, 1602.

[15] H. Gupta, Y. Dahiya, H. K. Rathore, K. Awasthi, M. Kumar, D. Sarkar, *ACS Appl. Mater. Interfaces* **2023**, *15*, 42685.

[16] Z. Pan, Z. Lu, L. Xu, D. Wang, *Appl. Surf. Sci.* **2020**, *510*, 145384.

[17] H. Fan, X. Hu, S. Zhang, Z. Xu, G. Gao, Y. Zheng, G. Hu, Q. Chen, T. S. AlGarni, R. Luque, *Carbon* **2021**, *180*, 254.

[18] B. Xue, J. Xu, R. Xiao, *Chem. Eng. J.* **2023**, *454*, 140192.

[19] H. Fan, S. Zhou, Q. Li, G. Gao, Y. Wang, F. He, G. Hu, X. Hu, *J. Colloid Interface Sci.* **2021**, *600*, 681.

[20] J. Wang, Y. Huang, X. Han, Z. Li, S. Zhang, M. Zong, *Appl. Surf. Sci.* **2022**, *579*, 152247.

[21] D. Wang, S. Wang, J. Sun, *Biomass Convers. and Biorefin.* **2024**, *14*, 7031.

[22] X. Deng, J. Li, Z. Shan, J. Sha, L. Ma, N. Zhao, *J. Mater. Chem. A* **2020**, *8*, 11617.

[23] H. Zhang, Q. Liu, Y. Fang, C. Teng, X. Liu, P. Fang, Y. Tong, X. Lu, *Adv. Mater.* **2019**, *31*, 1904948.

[24] Y. Yang, D. Chen, H. Wang, P. Ye, Z. Ping, J. Ning, Y. Zhong, Y. Hu, *Chem. Eng. J.* **2022**, *431*, 133250.

[25] J. Yu, L. Wang, J. Peng, X. Jia, L. Zhou, N. Yang, L. Li, *Ionics* **2021**, *27*, 4495.

[26] Y. Zhao, H. Hao, T. Song, X. Wang, C. Li, W. Li, *J. Power Sources* **2022**, *521*, 230941.

[27] L. Dong, X. Ma, Y. Li, L. Zhao, W. Liu, J. Cheng, C. Xu, B. Li, Q. H. Yang, F. Kang, *Energy Storage Mater.* **2018**, *13*, 96.

[28] X. Zhang, E. Cao, Y. Tian, M. Zhang, X. Liu, Z. Lei, Z. Zhao, P. Cui, Q. Ling, R. Xie, *Carbon Resour. Convers.* **2022**, *5*, 193.

[29] P. Song, C. Li, N. Zhao, Z. Ji, L. Zhai, X. Shen, Q. Liu, *J. Colloid Interface Sci.* **2023**, *633*, 362.

[30] X. Hou, P. Ren, W. Tian, R. Xue, B. Fan, F. Ren, Y. Jin, *J. Power Sources* **2024**, *603*, 234408.

[31] F. Kang, Y. Li, Z. Zheng, X. Peng, J. Rong, L. Dong, *J. Colloid Interface Sci.* **2024**, *669*, 766.

[32] X. Zhang, M. S. Javed, X. Zhang, S. Ali, K. Han, A. Ahmad, I. Hussain, A. M. Tighezza, W. U. Arifeen, W. Han, *J. Energy Storage* **2024**, *86*, 111208.

[33] J. Hong, C. Jo, *J. Power Sources* **2024**, *594*, 234006.
